# Associations among impostor phenomenon, self-compassion, shame, and social anxiety in college students

**DOI:** 10.3389/fpsyg.2026.1777854

**Published:** 2026-04-15

**Authors:** Chuanchuan He, Lin Ye

**Affiliations:** Office of Academic Affairs, Yunnan Minzu University, Kunming, Yunnan, China

**Keywords:** college students, imposter phenomenon, self-compassion, shame, social anxiety

## Abstract

**Background:**

Social anxiety represents a common internalizing emotional issue among college students, while the imposter phenomenon, as a negative self-cognitive pattern, is regarded as a key psychological factor associated with the triggering and sustaining of social anxiety. Although previous research has preliminarily explored the relationship between the two, the associations among these variables through which self-conscious emotions operate within this association remain insufficiently explained. Grounded in the Cognitive-Affective System Theory of Personality, this study systematically examines the relationships between imposter phenomenon, self-compassion, shame, and social anxiety.

**Methods:**

Using a stratified random sampling approach, the study conducted an online questionnaire survey across multiple universities, obtaining 1408 valid responses. The study conducted regression analyses using SPSS.

**Results:**

Hierarchical regression analysis revealed that when self-compassion and shame were entered into the regression models, impostor phenomenon was negatively associated with self-compassion and positively associated with shame and social anxiety. Self-compassion was negatively associated with both shame and social anxiety, while shame was positively associated with social anxiety. Moreover, gender significantly moderates the relationship between impostor phenomenon and both self-compassion and social anxiety.

**Discussion:**

By shedding new light on the complex relationship between the imposter phenomenon and social anxiety, these findings may inform student mental health support in college students.

## 1 Introduction

Social anxiety (SA) refers to a persistent, irrational psychological state of fear that arises in social or performance situations due to an individual’s apprehension of being scrutinized or negatively evaluated by others (Morrison and Heimberg, 2013). The college student population commonly experiences social comparison and is in a developmental stage of self-identity formation, a psychological condition that readily fosters the emergence of SA. Empirical data corroborates this: one study indicated that nearly one-third (33.38%) of college students endorsed at least one symptom of SA (Meng et al., 2021). Extensive research demonstrates that SA significantly reduces students’ willingness to participate and the quality of their performance in key learning activities such as group collaboration and class presentations, thereby limiting their academic achievement (Guo S. Q. et al., 2025; Sánchez-Vincitore et al., 2025). Crucially, if not effectively supported during this critical developmental period, SA in college students may intensify and subsequently increase their risk of social isolation, while also potentially evolving into psychological distress such as depression, rumination, and loneliness, which could create lasting obstacles to their career development and long-term social integration (Birg et al., 2025; Liu and Baharudin, 2025; Öztekin et al., 2025). College students, in a critical period of identity formation and heightened sensitivity to external evaluations, often experience the imposter phenomenon, where the dread of being exposed intensifies their fear of negative evaluation and may heighten SA (Fatemah et al., 2025). Concurrently, a scoping review indicates a significant negative correlation between self-compassion and SA, highlighting the potential role of psychological interventions based on self-compassion for this population (Slivjak et al., 2024). Shame, as a core emotional experience of the self-evaluative system, strengthens the avoidance and fear in social situations, thereby increasing the propensity for SA (Carpenter et al., 2022). Consequently, systematically investigating the correlates of SA among college students holds significant practical importance for improving their social functioning and promoting mental health.

While existing research has preliminarily revealed certain associations between imposter phenomenon, self-compassion, shame, and SA, there remains substantial room for further elaboration and expansion. First, prior studies have predominantly focused on examining the direct relationship between the imposter phenomenon and SA, with most analyses limited to their inverse correlation. A systematic investigation into potential positive or complex interactive pathways is notably lacking. Second, the way in which self-compassion and shame are correlated with both imposter phenomenon and SA, and whether these correlations are independent of each other, has not yet been systematically examined or theoretically integrated within college students. Therefore, this study aims to systematically examine the association connecting college students’ imposter phenomenon and SA, paying particular attention to the distinct roles of self-compassion and shame as correlates. The findings offer important theoretical and empirical insights for psychological interventions targeting college students and provide valuable implications for enhancing their self-acceptance and improving social adaptive functioning.

## 2 Review and hypothesis

### 2.1 Theoretical basis

The Cognitive-Affective System Theory of Personality (CAPS) posits that an individual’s behavior and psychological state in a specific situation are determined by their internal cognitive-affective interaction patterns (Mischel and Shoda, 1995). When individuals are fixated on a core belief of personal inadequacy, they tend to adopt an excessively critical attitude toward themselves. This persistent self-negation can readily transform momentary self-doubt into pervasive feelings of shame, which may ultimately manifest as intense anxiety in social situations that require a demonstration of competence. Specifically, a high level of imposter phenomenon is strongly associated with low self-compassion, which manifests as the lack of self-kindness and understanding along with the tendency toward self-criticism in achievement-related situations (Bender-Burnett, 2025). This deficit in self-compassion is further linked to pronounced feelings of shame, as individuals tend to attribute temporary setbacks to a globally flawed self, thereby reinforcing a negative identity rooted in perceived undeservedness (Shamsababdi and Dehshiri, 2024). The internal pressure fueled by this self-criticism and shame can ultimately translate into an ongoing dread of negative appraisal and hyper-vigilance within social contexts, contributing to the development of SA (Huang et al., 2024). This proposed pattern of association aligns theoretically with the perspective of CAPS, illustrating how different cognitive and affective units can be activated by situations and interconnect to shape specific behaviors and psychological states. Grounding its framework in CAPS, this study systematically proposes a theoretical model. This model aims to delineate how imposter phenomenon, as a deep-seated cognitive-personality structure, is theoretically associated with SA among college students in relation to self-compassion and shame.

### 2.2 Impostor phenomenon and social anxiety

Impostor phenomenon refers to the psychological experience where individuals persistently believe they are incompetent, attribute their success to external factors, and fear being exposed as frauds despite objective achievements (Clance and Imes, 1978). According to the CAPS (Mischel and Shoda, 1995), once the negative self-schema inherent in the impostor phenomenon is activated in social situations, individuals tend to experience persistent fear of negative evaluation along with intense anxiety. This cognitive-affective pathway may be particularly pronounced in collectivist cultural contexts such as China, where self-worth is closely tied to social evaluation and group harmony. Under cultural norms that emphasize evaluative feedback and face, the fear of being exposed as incompetent, a core feature of the impostor phenomenon, is amplified, leading to heightened anxiety in evaluative social contexts (Li, 2026). Prior research has documented significant positive associations between the impostor phenomenon and various forms of anxiety-related constructs, including general anxiety (Brauer, 2026), communication anxiety (Brauer et al., 2023a), SA (Fatemah et al., 2025), fear of negative evaluation (Brauer and Wolf, 2016). Individuals with more severe impostor feelings typically perceive higher levels of pressure and anxiety (Jansson et al., 2025), but are also characteristically marked by a heightened fear of being laughed at or humiliated by others (Brauer and Proyer, 2023). College students, who face evaluative pressure and identity transition, are more likely to experience psychological avoidance and behavioral inhibition in social situations such as class presentations, group discussions, and seeking help due to the impostor phenomenon (Shin and Lytle, 2024). Long-term avoidance of social interaction may weaken actual social skills and self-confidence, further reinforcing negative self-perceptions of inadequacy and creating a vicious cycle of heightened anxiety and behavioral withdrawal (Ernst et al., 2023). Therefore, a deeper investigation into the association between impostor phenomenon and SA can help college students with strong impostor feelings break this cycle and establish more positive and stable self-perceptions, thereby promoting healthier and more adaptive development in both academic and social contexts.

### 2.3 Impostor phenomenon, self-compassion, and social anxiety

Self-Compassion is defined as the capacity to treat themselves with warmth, understanding, and acceptance when facing personal failures, shortcomings, or suffering, rather than with excessive criticism or avoidance (Neff K., 2003; Neff K. D., 2003). According to the CAPS (Mischel and Shoda, 1995), when individuals experiencing impostor phenomenon enter social situations where evaluation is possible, their core cognitive schema of inadequacy and the affective unit associated with fear of failure are activated. This activation makes it difficult for them to engage in self-compassion, thereby heightening their anxiety about potential negative evaluation from others and creating a persistent negative cycle. Empirical research has shown a significant negative association between impostor phenomenon and self-compassion (Kornsawad et al., 2025). For example, a quantitative analysis by Bender-Burnett (2025) found a significant negative association between the severity of the impostor phenomenon and self-compassion in student populations, indicating that individuals with stronger impostor phenomenon possess a weaker capacity to treat their own shortcomings and failures with kindness, understanding, and acceptance. Similarly, a longitudinal study of first-year medical students confirmed that those reporting more severe impostor phenomenon typically exhibited lower self-compassion and greater difficulty in self-soothing and self-understanding when confronted with setbacks (Rosenthal et al., 2021). These studies collectively indicate that impostor phenomenon significantly impairs self-compassion, and the absence of this self-soothing and self-accepting emotional capacity forms the core cognitive and affective foundation for individual’s tendency to avoid social interactions.

Existing studies consistently support a significant negative association between self-compassion and SA (Cutajar and Bates, 2025). Specifically, individuals with higher self-compassion tend to more effectively engage positive self-dialog and emotion regulation strategies when facing evaluative pressure in social situations, thereby significantly alleviating anxiety experiences (McBride et al., 2022). For instance, research by Holas et al. (2023) confirmed that enhancing an individual’s level of self-compassion effectively reduces their degree of SA. Similarly, Sapach and Carleton (2023) noted that systematic training centered on self-compassion can serve as an effective intervention pathway for alleviating SA. Furthermore, a study by Gao et al. (2023) focusing on Chinese college students found that self-compassion is closely associated with both appearance anxiety and SA, further highlighting its protective function against SA. This protective function takes on added significance within China’s distinct socio-cultural context. In a collectivist society where self-worth is heavily influenced by external evaluation, diminished self-compassion renders college students more vulnerable to social feedback and less capable of mitigating self-criticism in interpersonal settings, thereby intensifying their SA (Wang et al., 2025). This collective evidence indicates that self-compassion may serve as an important psychological factor closely associated with both the impostor phenomenon and SA. A deeper exploration of these relations would offer a more integrated theoretical perspective for explaining the formation of SA, and could provide clear empirical foundations and feasible intervention targets for psychological interventions aimed at individuals with high levels of the impostor phenomenon.

### 2.4 Impostor phenomenon, shame, and social anxiety

Shame is a painful self-conscious emotion arising from the perception that one’s entire self is fundamentally flawed or has violated personal standards, often accompanied by feelings of exposure and a tendency to withdraw (Gilbert, 2003). From the perspective of CAPS (Mischel and Shoda, 1995), the ingrained fear of exposure associated with the impostor phenomenon consistently activates an individual’s shame regarding the revelation of self-perceived flaws, which directly translates into anxiety in response to others’ evaluations within social contexts. Existing research confirms that individuals with high levels of the impostor phenomenon commonly report experiencing more intense shame (Hudson and González-Gómez, 2021). This association is particularly pronounced among college students, who are in a critical stage of transition between academic achievements and social roles. For this population, impostor phenomenon deepens shame by fueling persistent concerns about inadequacy and potential failure in evaluative settings, an inherently social response tied to unmet expectations of significant others in collectivist cultures (Hu et al., 2019). This indicates that shame could be a crucial psychological factor that co-occurs with impostor phenomenon and is closely related to emotional distress in this group.

A systematic review integrating 60 eligible empirical studies concluded that a robust positive association exists between shame and SA, with preliminary evidence suggesting this relationship holds across different cultural contexts and clinical populations (Swee et al., 2021). For example, the study focusing on children and adolescents further confirmed a significant positive relationship between experiences of shame and SA in this group (Ahandany et al., 2024), indicating that this association emerges early in individual development. More importantly, intervention-based evidence shows that targeted interventions addressing shame can effectively alleviate an individual’s SA (Wen et al., 2024). Building on these established links, numerous studies have further revealed the close associations among shame, various social risk factors, and SA (Bardezard et al., 2025; Wu et al., 2021). The collective evidences demonstrate that shame is not merely a correlate of SA but may be a key factor that is concurrently associated with both the impostor phenomenon and SA. This provides a clear theoretical entry point and a clinical intervention direction in understanding and addressing SA among college students.

### 2.5 Self-compassion and shame

Prior study has demonstrated the significant inverse association between self-compassion and shame (Shamsababdi and Dehshiri, 2024). For example, Siwik et al. (2022) found that higher self-compassion typically correspond to lower experiences of shame. Concurrently, self-compassion interventions have proven effective in alleviating an individual’s shame (Fink-Lamotte et al., 2023). While existing research have revealed the independent associations of both self-compassion and shame with impostor phenomenon and SA respectively, the relationships among these variables have not yet been systematically explored. Grounded in the framework of Conservation of Resources theory (Hobfoll, 1989), an individual’s capacity for self-compassion can be conceptualized as a psychological resource that buffers against psychological stress. This resource can protect individuals from resource loss when facing threatening stimuli. From this perspective, the impostor phenomenon may be associated with a depletion of this resource, and such resource loss may, in turn, be linked to heightened experiences of shame. This is indirectly supported by recent empirical work. For instance, a study among secondary school students found that self-compassion and shame were closely interrelated with parental conflict and SA (Huang et al., 2024). This finding provides preliminary empirical support for the involvement of self-compassion and shame in the psychological adaptation process.

### 2.6 The moderating role of gender

Previous research has indicated that there are gender differences in impostor phenomenon, self-compassion, and SA (Guo S. P. et al., 2025; Price et al., 2024; Rowlinson et al., 2026). Jansson et al. (2025) further revealed that female college students reported higher mean scores than their male counterparts for impostor phenomenon, and also scored significantly higher on measures of anxiety. Research by Rosenthal et al. (2021) also demonstrated that students experiencing severe impostor phenomenon typically report lower self-compassion, with notable gender differences observed in this relationship. Therefore, this study included gender as a moderating variable to examine its role in the associations between the impostor phenomenon and both self-compassion and SA.

### 2.7 Hypothesis

Building upon the relevant literature and prior empirical findings, the following hypotheses are proposed:

*H1*: Impostor phenomenon is positively correlated with SA among college students.

*H2*: Impostor phenomenon is negatively correlated with self-compassion.

*H3*: Self-compassion is negatively correlated with SA.

*H4*: Impostor phenomenon is positively correlated with shame.

*H5*: Shame is positively correlated with SA.

*H6*: Self-compassion is negatively correlated with shame.

*H7*: Gender significantly moderates the relationship between impostor phenomenon and SA.

*H8*: Gender significantly moderates the relationship between impostor phenomenon and self-compassion.

## 3 Research method

### 3.1 Sample and data collection

Data collection for this study was conducted online via the digital questionnaire platform www.Sojump.com. The survey was administered between October and December 2025, targeting enrolled college students at three universities in Kunming, Yunnan Province: Yunnan Minzu University, Yunnan University of Finance and Economics, and Yunnan Agricultural University. To ensure the representativeness of the sample, a stratified random sampling method was adopted. First, within the selected universities, stratification was carried out by grade level (freshman to senior year), and several classes were randomly sampled from each grade. Subsequently, questionnaire links were sent via the university’s academic affairs system or class contact groups to all students in the selected classes. This method ensured that, at the grade level, each student had a known and non-zero probability of being selected due to the random selection of their class, thereby meeting the requirements of probability sampling. This allowed the sample to effectively reflect the overall distribution of students across various grade levels. The study protocol was approved by Yunnan Minzu University, and all procedures complied with guidelines for research involving human participants. Participation was entirely voluntary, and all participants provided informed consent after being fully informed about the study content in the pre-questionnaire stage. The study was conducted in full compliance with ethical standards. Participants’ privacy and autonomy were rigorously protected throughout.

An *a priori* Monte Carlo power analysis for the hypothesis model (Schoemann et al., 2017) was conducted to determine the necessary sample size. Assuming small-to-medium effect sizes, the analysis suggested that a minimum of 445 participants was required to achieve sufficient power (0.80) to detect the hypothesized effects at an alpha level of 0.05. During the actual data collection process, 1,487 questionnaires were distributed and all were returned. Strict screening was applied to the returned questionnaires. The data cleaning process followed these criteria: (1) Valid questionnaires were required to have at least 80% completion rate; questionnaires with missing values exceeding 20% were considered invalid. For questionnaires with partial missing data that did not meet the exclusion threshold, multiple imputation was used to handle the missing values. (2) Questionnaires displaying obvious extreme response patterns, such as answers concentrated exclusively on “strongly agree” or “strongly disagree” options, were excluded. (3) Following the approach of Meade and Craig (2012), the 3rd percentile of the total response time (90 s) was used as an absolute threshold to exclude fast responders whose completion time fell below this threshold. Ultimately, 1,408 valid questionnaires were retained, which significantly exceeded the requisite sample size, yielding a valid response rate of 94.69%. As shown in Table 1, the sample demonstrated reasonable distribution across key demographic variables, indicating suitability for subsequent analyses.

**Table 1:** Demographic characteristics of the sample.

Demographic characteristics	Category	Amount	Percentage (%)
Gender	Men	669	47.5
	Women	739	52.5
Age	18–19	516	36.7
	20–21	465	33.0
	≥22	427	30.3
Grade	Freshman	450	32.0
	Sophomore	432	30.7
	Junior	323	22.9
	Senior	203	14.4
Household registration location	Cities and towns	663	47.1
	Village	745	52.9

### 3.2 Measurement instruments

To ensure the applicability and validity of the scales used in the context of Chinese culture, the scales measuring self-compassion, shame, and SA in this study were all in Chinese versions, which have been validated and proven to have good adaptability. For the impostor phenomenon scale, the following steps were taken for cultural adaptation. First, a bilingual psychology expert independently translated the scale from English to Chinese. Then, another expert who had not been exposed to the original version back-translated the Chinese version into English and compared it with the original version, revising any ambiguous items until the Chinese and English versions were semantically equivalent. Next, a cultural psychology expert and five Chinese college students were invited to review the phrasing of the Chinese version, assessing whether it was clear and easy to understand in the Chinese context. Based on their feedback, certain words were localized. Prior to formal administration, we conducted a pre-test with 50 college students, and the scale demonstrated good reliability and validity in the sample, indicating strong psychometric properties.

The Clance Impostor Phenomenon Scale, developed by Clance (1985), was used in this study to assess impostor phenomenon among college students. The scale comprises 20 items (e.g., “Sometimes you feel your success is due to a kind of luck”), which measure three dimensions: self-doubt regarding one’s intelligence and ability, a tendency to attribute success to luck, and difficulty internalizing accomplishments. Responses are rated on a 5-point Likert scale, with higher scores indicating more severe impostor phenomenon. This scale has been validated for use in Chinese cultural contexts and has demonstrated applicability among college student populations (Wei et al., 2024). This three-factor first-order model demonstrates a satisfactory overall fit: *χ*^2^/df = 2.639, GFI = 0.977, IFI = 0.989, CFI = 0.989, NFI = 0.982, RFI = 0.972, TLI = 0.982, RMR = 0.017, RMSEA = 0.034.

This study used the Self-Compassion Scale, originally developed by Neff K. (2003) and Neff K. D. (2003) and later adapted by Gong et al. (2014), to measure self-compassion among college students. The scale consists of 12 items (e.g., “When feeling depressed, you try to adjust your emotions with a positive and open-minded attitude”), which assess three dimensions: self-kindness, common humanity, and mindfulness. Responses are recorded on a 5-point Likert scale, and certain items required reverse scoring prior to analysis (items 2, 4, 5, 8, 11). Elevated total scores are indicative of higher self-compassion. This scale has been validated for use in Chinese cultural contexts and has demonstrated applicability among college student populations (Ma and Xiao, 2024). The scale demonstrates a satisfactory overall fit: *χ*^2^/df = 2.695, GFI = 0.989, IFI = 0.993, CFI = 0.993, NFI = 0.989, RFI = 0.981, TLI = 0.988, RMR = 0.009, RMSEA = 0.035.

The Shame Scale, developed by Qian (2000), was employed in this study to assess college students’ shame across three dimensions: personality, behavior, and physique. The scale consists of 25 items (e.g., “Have you ever felt shame because you did something wrong?”) and uses a 4-point Likert scale (1 = Never, 4 = Frequently). Higher scores indicate greater levels of shame. The reliability and validity of this scale for measuring shame in Chinese college students have been supported by previous research (Ye and Su, 2025). The scale demonstrates a satisfactory overall fit: *χ*^2^/df = 2.612, GFI = 0.973, IFI = 0.989, CFI = 0.989, NFI = 0.982, RFI = 0.970, TLI = 0.981, RMR = 0.013, RMSEA = 0.034.

The Social Anxiety Scale, originally developed by Leary (1983) and later revised by Li et al. (2024), was used in this study to assess the level of SA among college students. The scale consists of 13 items (e.g., “You rarely feel anxious in social situations”), measuring two dimensions: tension and relaxation. All items were measured on a 5-point Likert scale. Items 3, 8, and 13 are reverse-scored, with higher total scores indicating higher levels of SA. The reliability and validity of this scale for measuring SA in Chinese college students have been supported by previous research (Zhu et al., 2025). The scale demonstrates a satisfactory overall fit: *χ*^2^/df = 2.812, GFI = 0.986, IFI = 0.993, CFI = 0.993, NFI = 0.989, RFI = 0.982, TLI = 0.988, RMR = 0.016, RMSEA = 0.036.

### 3.3 Statistical analysis

Data analysis was conducted using SPSS 27.0 and AMOS 28.0. First, descriptive statistical analysis was conducted to examine the basic distribution characteristics of each variable. Second, the reliability and validity of the scales used in the study were tested. On this basis, confirmatory factor analysis (CFA) was employed to assess the goodness of fit of the measurement model and to test for potential common method bias (CMB). Subsequently, hierarchical regression analysis was employed to examine the associations among variables. Finally, moderation effect analysis was conducted using the SPSS PROCESS macro (Version 4.2).

## 4 Results

### 4.1 Descriptive statistics and correlation test

To ensure multivariate assumptions were met and results were valid, the study checked data normality through skewness and kurtosis and calculated descriptive statistics. The absolute values of skewness and kurtosis in Table 2 did not exceed 2 and 7, respectively, meeting the requirements for normality (Kim, 2013).

**Table 2:** The description and correlation of variables.

Variables	*M* ± SD	Skewness	Kurtosis	Impostor phenomenon	Self-compassion	Shame	SA
Impostor Phenomenon	3.119 ± 0.696	−0.200	−0.477	1			
Self-Compassion	3.420 ± 0.538	−0.215	0.032	−0.441***	1		
Shame	2.249 ± 0.576	0.167	−0.471	0.565***	−0.456***	1	
SA	3.261 ± 0.718	−0.014	−0.047	0.502***	−0.426***	0.528***	1

*N* = 1,408, significance: ***p < 0.001. M, mean; SD, standard deviation; SA, social anxiety, the same applies below.

The correlation analysis results presented in Table 2 indicated a significant negative correlation between impostor phenomenon and self-compassion (*r* = −0.441, *p* < 0.001). Impostor phenomenon was significantly positively correlated with shame (*r* = 0.565, *p* < 0.001) and SA (*r* = 0.502, *p* < 0.001). Self-compassion was significantly negatively correlated with shame (*r* = −0.456, *p* < 0.001) and SA (*r* = −0.426, *p* < 0.001). Shame showed a significant positive correlation with SA (*r* = 0.528, *p* < 0.001). Overall, the directions of the relationships among the variables were consistent with theoretical predictions.

### 4.2 Reliability and validity testing

To comprehensively assess the stability and validity of the measurement instruments, a systematic evaluation of scale reliability and validity was conducted. Regarding reliability, the Cronbach’s alpha and composite reliability (CR) values in Table 3 for all latent variables exceeded 0.9, surpassing the recommended threshold of 0.7, indicating excellent internal consistency reliability (Hair et al., 2021). In terms of validity, the average variance extracted (AVE) for each latent variable was greater than 0.5, meeting the suggested criterion and confirming good convergent validity (Henseler et al., 2015). All heterotrait–monotrait ratios (HTMT) in Table 4 were well below 0.85, and the square root of the AVE for each latent variable was significantly greater than its correlation coefficients with other variables in Table 5, which collectively confirm that the scales possess good discriminant validity (Hair et al., 2021).

**Table 3:** The results of reliability and validity tests.

Variables	Cronbach’s alpha	CR	AVE
Impostor Phenomenon	0.956	0.960	0.543
Self-Compassion	0.923	0.934	0.542
Shame	0.965	0.968	0.546
SA	0.937	0.945	0.570

**Table 4:** The results of HTMT.

Variables	Impostor phenomenon	Self-compassion	Shame	SA
Impostor Phenomenon				
Self-compassion	0.469			
Shame	0.588	0.480		
SA	0.534	0.461	0.559	

**Table 5:** The results of Fornell–Larcker criterion test.

Variables	Impostor phenomenon	Self-compassion	Shame	SA
Impostor Phenomenon	0.737			
Self-compassion	−0.442	0.736		
Shame	0.567	−0.454	0.739	
SA	0.514	−0.432	0.541	0.755

The square roots of the AVE values should be formatted in bold and italic and displayed on the diagonal of the table.

### 4.3 Common method bias

Self-reported data carry an inherent risk of CMB. For this purpose, Harman’s single-factor test was initially employed for preliminary analysis. Exploratory factor analysis results indicated the extraction of eight factors with eigenvalues greater than 1. The first factor did not exceed the 40% critical threshold, explaining 35.442% of the variance (Podsakoff and Organ, 1986), providing initial evidence that CMB was not a substantial concern. To further enhance the robustness of this conclusion, the Unmeasured Latent Method Factor approach was subsequently applied for testing (Podsakoff et al., 2003). First, CFA was conducted on the measurement model to ensure the validity of the latent variable measurements, establishing it as the baseline model. Based on this, a common method factor was added to the baseline model, with its factor loadings on all items constrained to be equal, to construct a controlled model. By comparing the fit of the two models, the presence of significant CMB can be detected.

Given the characteristics of ordinal categorical data, methodological literature typically recommends employing robust estimation methods, such as weighted least squares mean and variance adjusted (WLSMV) or maximum likelihood estimation with robust standard errors (MLR) (Brauer et al., 2023b; Li, 2016). Given that the analytical tool for this study is AMOS software, its default estimation method is Maximum Likelihood (ML). To verify the applicability of this method, we tested the data distribution. The results indicated that the skewness and kurtosis of each measurement item fell within an acceptable range approximating a normal distribution (see Table 2). Under these conditions, even with ordinal categorical data, using ML estimation can generally yield relatively robust parameter estimates (Kline, 2023). Nevertheless, to further control for potential standard error estimation bias arising from non-normality, we employed the bias-corrected percentile Bootstrap method (with 5,000 resamples). This method does not rely on strict normality assumptions and can generate more robust confidence intervals (Preacher and Hayes, 2008). As shown in Table 6, both the baseline model and the controlled model demonstrated good overall goodness-of-fit (Hu and Bentler, 1998). The criteria for judging model differences comprehensively referred to methodological literature on measurement invariance testing (Chen, 2007; Cheung and Rensvold, 2002), and all observed differences were well below these statistical thresholds. This result indicates that introducing the common method factor did not lead to a substantial improvement in model fit, suggesting that no significant CMB was detected. This provides more robust statistical support for the preliminary conclusion drawn from the aforementioned Harman’s single-factor test.

**Table 6:** The comparison results between the baseline model and the control model.

Model	*χ*^2^/df	RMSEA	SRMR	GFI	NFI	CFI	IFI	TLI	RFI
Reference value	<3	<0.06	<0.06	>0.9	>0.9	>0.9	>0.9	>0.9	>0.9
Baseline model	1.683	0.025	0.034	0.929	0.948	0.978	0.978	0.975	0.941
Control model	1.684	0.025	0.034	0.929	0.948	0.978	0.978	0.975	0.941
Difference value	0.001	<0.001	<0.001	<0.001	<0.001	<0.001	<0.001	<0.001	<0.001
Judgment standard		Δ < 0.01	Δ < 0.025	Δ < 0.01	Δ < 0.01	Δ < 0.01	Δ < 0.01	Δ < 0.02	Δ < 0.01

### 4.4 Collinearity test

To ensure robust and reliable conclusions, variance inflation factor (VIF) was employed to diagnose and assess multicollinearity among variables. All values of VIF in Table 7 were below 3.3, ruling out significant multicollinearity concerns (Hair et al., 2019).

**Table 7:** The results of collinearity test.

Variables	Impostor phenomenon	Self-compassion	Shame	SA
Impostor phenomenon				
Self-compassion	1.000			
Shame	1.242	1.242		
SA	1.573	1.345	1.595	

### 4.5 Hierarchical regression analysis

Following the analytical approach and effect size benchmarks suggested by Keith (2019), hierarchical regression analysis was employed to examine the associations among impostor phenomenon, self-compassion, shame, and SA. The results are shown in Table 8. Impostor phenomenon was negatively associated with self-compassion. The model was significant, explaining 19.4% of the variance in self-compassion (adjusted *R*^2^ = 0.194). With shame as the dependent variable, impostor phenomenon was entered in the first step, and self-compassion was added in the second step. Impostor phenomenon alone explained 31.9% of the variance in shame (adjusted *R*^2^ = 0.318). Adding self-compassion increased this to 37.2% (adjusted *R*^2^ = 0.371), with both models significant. Specifically, impostor phenomenon was positively associated with shame, self-compassion was negatively associated with shame, and self-compassion contributed an incremental explanatory rate of 5.3% to shame. With SA as the dependent variable, impostor phenomenon was entered in the first step, self-compassion was entered in the second step, and shame was entered in the third step. Impostor phenomenon alone explained 25.2% of the variance in SA (adjusted *R*^2^ = 0.251). Adding self-compassion increased this to 30.4% (adjusted *R*^2^ = 0.303), with both models significant. After adding shame, the model remained significant, explaining 36.3% of the variance in SA (adjusted *R*^2^ = 0.362). Self-compassion and shame contributed incremental explanatory rates of 5.2 and 5.9% to SA, respectively. Specifically, impostor phenomenon and shame were positively associated with SA, whereas self-compassion was negatively associated with SA. These cross-sectional associations are consistent with the proposed hypotheses, but causal ordering cannot be determined.

**Table 8:** The results of regression analysis.

Dependent variable	Independent variable	*β*	*T*	*p*	*F*	*R* ^2^	Adjusted *R*^2^
Self-compassion	Impostor phenomenon	−0.441	−18.411	<0.001	338.959***	0.194	0.194
Shame	Impostor phenomenon	0.565	25.644	<0.001	657.590***	0.319	0.318
	Impostor phenomenon	0.451	19.162	<0.001	415.610***	0.372	0.371
	Self-compassion	−0.257	−10.891	<0.001			
SA	Impostor phenomenon	0.502	21.762	<0.001	473.588***	0.252	0.251
	Impostor phenomenon	0.390	15.730	<0.001	306.692***	0.304	0.303
	Self-compassion	−0.254	−10.238	<0.001			
	Impostor phenomenon	0.252	9.443	<0.001	266.642***	0.363	0.362
	Self-compassion	−0.175	−7.090	<0.001			
	Shame	0.307	11.409	<0.001			

### 4.6 Analysis of moderating effects

Table 9 presents the results of the moderation analysis. The interaction term between impostor phenomenon and gender was significantly negatively correlated with self-compassion (*β* = −0.160, *p* = 0.001) and significantly positively correlated with SA (*β* = 0.106, *p* = 0.017). This indicates that gender plays a significant moderating role in the relationship between impostor phenomenon and both self-compassion and SA. H7 and H8 were all supported.

**Table 9:** The moderation effect of gender.

Outcome variable	Predictor variable	*β*	*p*	SE	*T*	95% CI	
						LLCI	ULCI
Self-compassion	Impostor phenomenon × Gender	−0.160	0.001	0.050	−3.230**	−0.257	−0.063
SA	Impostor phenomenon × Gender	0.106	0.017	0.044	2.976*	0.019	0.192

**Figure 1:**
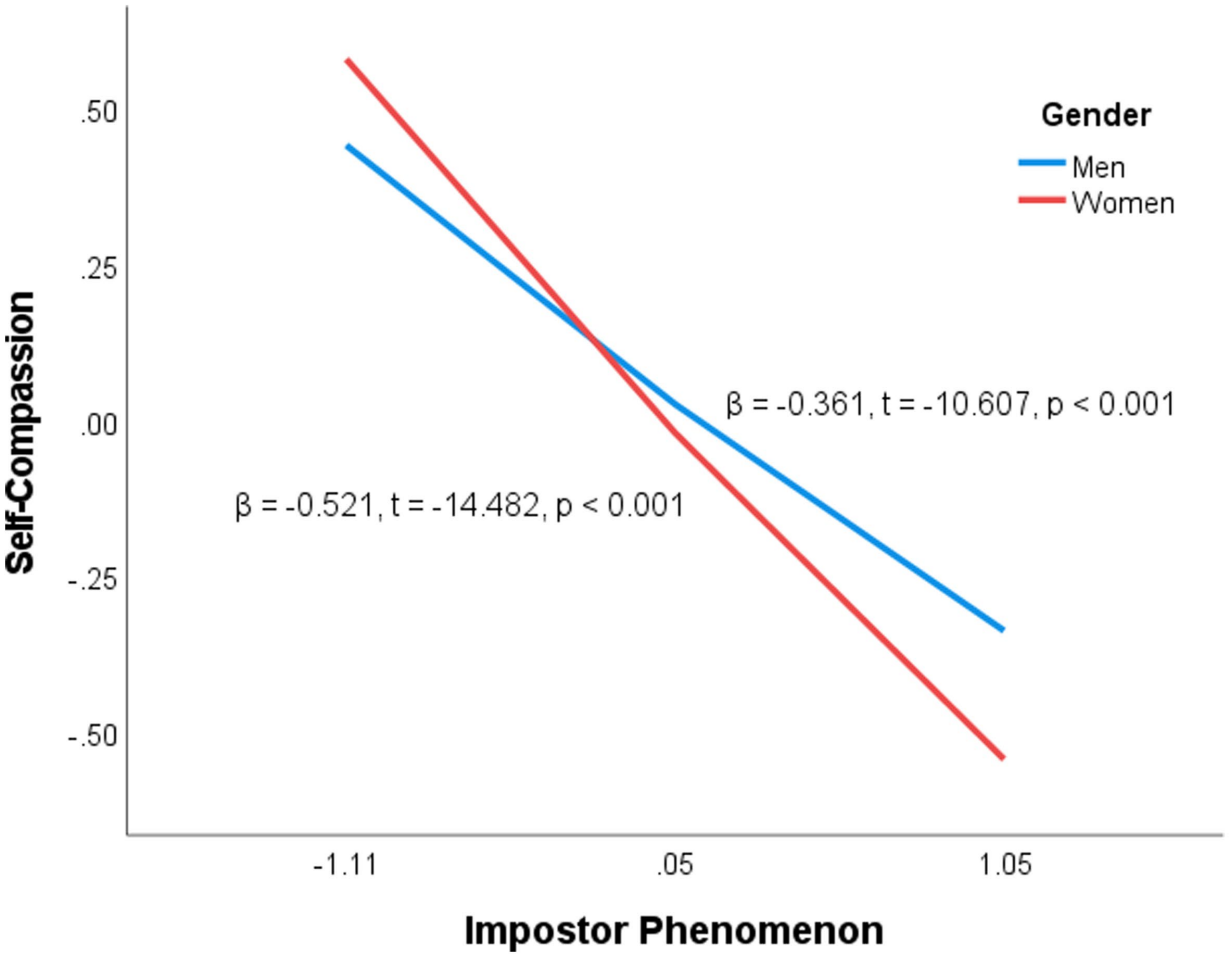
Moderating effect of gender on the association between impostor phenomenon and self-compassion.

**Figure 2:**
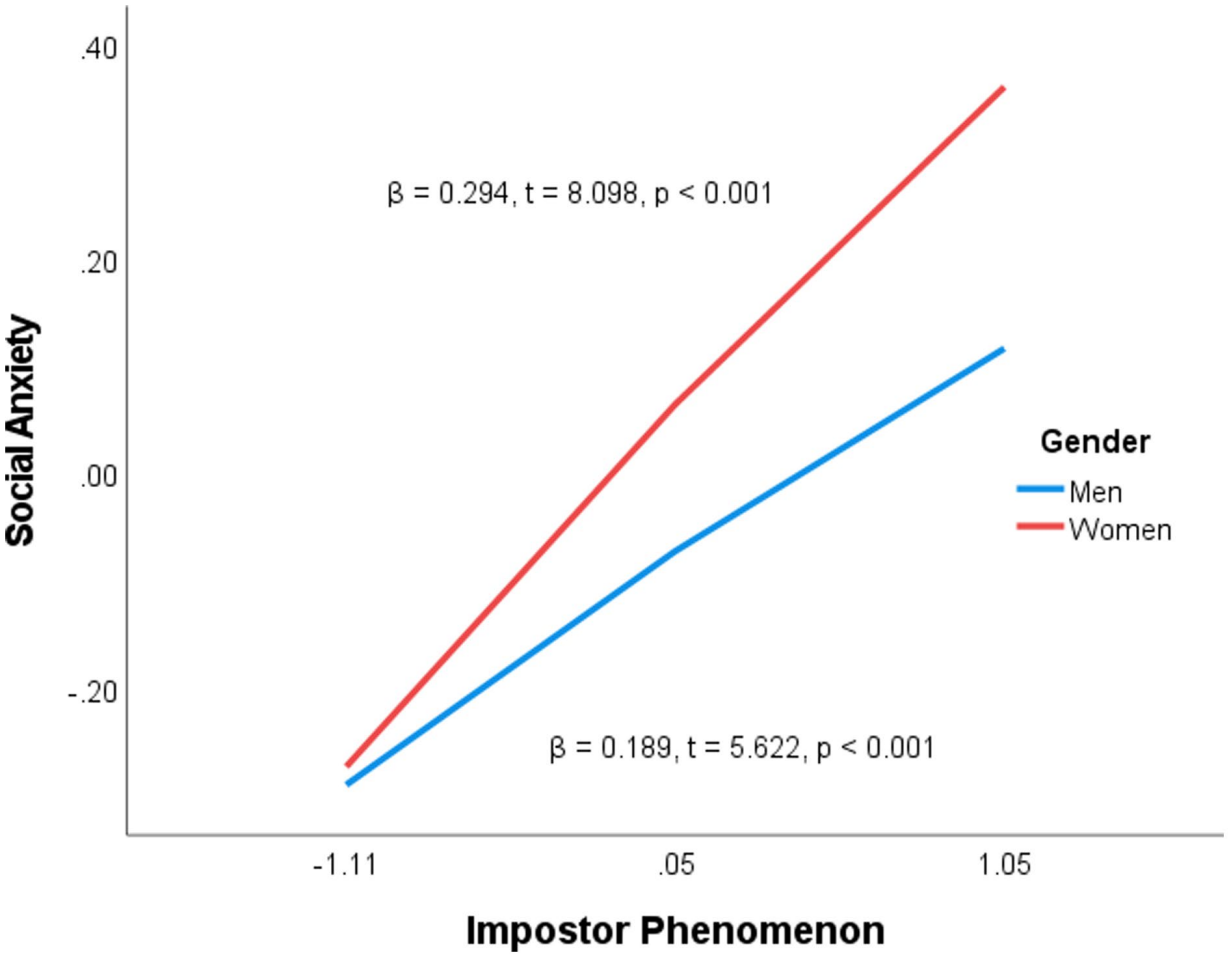
Moderating effect of gender on the association between impostor phenomenon and SA.

The simple slope analysis charts (see Figures 1, 2) reveal that the slopes for women are steeper than those for men, suggesting that the negative correlation between impostor phenomenon and self-compassion, as well as the positive correlation between impostor phenomenon and SA, is stronger among women. In other words, as impostor phenomenon becomes more severe, women experience a greater decline in self-compassion and a corresponding increase in SA.

## 5 Discussion

The findings of this study indicate a significant positive association between the impostor phenomenon and SA among college students, meaning that individuals with more serious impostor phenomenon is linked to higher SA. This result aligns with the conclusions drawn by Fatemah et al. (2025). Within the framework of CAPS (Mischel and Shoda, 1995), the impostor phenomenon, as a persistent cognitive self-bias, increases the likelihood of SA in college students in that it functions by triggering and maintaining a series of negative internal emotions. Existing research notes that the impostor phenomenon is particularly prominent in the college student population, who often fear being exposed as incompetent, leading to noticeable anxiety and avoidance tendencies in evaluative social and academic contexts (Shin and Lytle, 2024). Furthermore, this phenomenon is not only prevalent across different genders and age groups but is also frequently comorbid with anxiety and depression, which further reduces engagement and performance in interpersonal interactions and academic work (Bravata et al., 2020; Guimaraes et al., 2025). Therefore, this research suggests that interventions for individuals struggling with the impostor phenomenon should not merely focus on alleviating surface-level anxiety symptoms. Instead, efforts should aim to correct the underlying cognitive bias regarding self-competence. Addressing this core cognitive distortion can reduce the fear induced by the phenomenon at its source, thereby lowering the risk of the development and persistence of SA.

The results of this study demonstrate a significant negative association between impostor phenomenon and self-compassion, as well as a significant negative association between self-compassion and SA. Hierarchical regression analysis further reveals that after including for self-compassion, the positive correlation between impostor phenomenon and social anxiety remained significant but showed a reduction in magnitude. This pattern of associations suggests that self-compassion is closely related to both impostor phenomenon and SA. These findings are consistent with existing literature. Bender-Burnett (2025) noted that individuals experiencing high levels of the impostor phenomenon often remain in a state of self-criticism and deficient self-compassion due to excessive focus on personal shortcomings and a strong fear of negative evaluation. Viewed through the CAPS, the impostor phenomenon represents a persistent negative self-cognitive pattern that inhibits an individual’s inherent tendency toward self-understanding and self-kindness, thereby reinforcing and intensifying the cycle of self-criticism and compassion deficiency (Rosenthal et al., 2021). Furthermore, research by McBride et al. (2022) indicates that individuals with lower self-compassion typically experience higher SA. Within the specific socio-cultural context of China, lower self-compassion makes college students more susceptible to external evaluations and less able to alleviate self-criticism and pressure during interpersonal interactions, thereby significantly exacerbating their SA (Wang et al., 2025). Building on this understanding, emerging evidence further suggests that the persistent self-doubt and fear of negative evaluation triggered by impostor phenomenon significantly impair the ability to engage in self-compassion (Kornsawad et al., 2025). Lower levels of self-compassion are, in turn, associated with weaker regulation of negative self-cognitions and emotional distress when facing evaluative social situations, which is linked to a higher risk of SA (Cutajar and Bates, 2025). Therefore, implementing interventions focused on enhancing self-compassion among college student populations holds promise for alleviating SA.

This study demonstrate the significant positive relationship between impostor phenomenon and shame, and similarly, a significant positive relationship between shame and SA. Hierarchical regression analysis further reveals that shame shows significant associations with both impostor phenomenon and SA. In other words, individuals with more severe impostor feelings tend to experience stronger shame, and these experiences of shame tend to co-occur with higher SA. This relationship aligns with the research conclusions of Hudson and González-Gómez (2021) and Swee et al. (2021). In social situations involving potential evaluation by others, high levels of the impostor phenomenon predispose individuals to activate core negative beliefs about being fundamentally flawed or undeserving of their achievements, thereby triggering intense shame (Hu et al., 2019). The persistent experience of shame can establish a self-reinforcing emotional cycle, increasing an individual’s vulnerability to social psychological pressure and ultimately manifesting as more pronounced SA (Carpenter et al., 2022). The CAPS (Mischel and Shoda, 1995) emphasizes that specific emotional states connect an individual’s cognitive patterns to their overt behavior and internal psychological experiences. From this theoretical perspective, the impostor phenomenon and shame are closely associated, and this co-occurrence is related to an individual’s risk of SA. Therefore, this study provides empirical support and a theoretical entry point for future targeted interventions focusing on the dual pathways of emotion regulation and cognitive restructuring.

Hierarchical regression analysis showed that over and above the contributions of self-compassion and shame, impostor phenomenon remained significantly positively correlated with SA, and self-compassion was negatively correlated with shame. Specifically, the impostor phenomenon is often accompanied by diminished self-compassion, forming an internal state diminished self-compassion detrimental to emotional regulation (Kornsawad et al., 2025). This insufficiency in self-compassion is closely associated with a deeper emotional experience of shame (Shamsababdi and Dehshiri, 2024), and this pattern of associations is related to increased psychological vulnerability during the cognitive appraisal and behavioral response processes in social situations, thereby becoming significantly associated with the occurrence and maintenance of SA (Ahandany et al., 2024). Interpreting this through the lens of the CAPS (Mischel and Shoda, 1995), the impostor phenomenon, as a persistent negative self-cognitive pattern, is associated with a depletion of an individual’s self-support resources for emotional regulation, manifesting as reduced self-compassion. This reduced availability of cognitive-affective resources is linked to shame, a self-focused negative emotional state, which in turn is associated with anxious behavioral and psychological reactions within social contexts. The study results demonstrate that impostor phenomenon and SA are not simply related in isolation; rather, they are closely intertwined with self-compassion and shame. The finding further refines the understanding of the interrelated factors at both cognitive and emotional levels in the context of SA.

Findings indicated that gender played a significant moderating role in the links from impostor phenomenon to self-compassion and to SA, and these effects were more pronounced for female college students. This finding aligns with the results of Patzak et al. (2017): women scored significantly higher than men on measures of the impostor phenomenon, while reporting lower levels of self-compassion. This gender difference may stem from women being more susceptible to the impostor phenomenon (Kristoffersson et al., 2024), which in turn undermines their objective perception and internal acknowledgment of their own abilities. However, a study by Fatemah et al. (2025) reached a different conclusion, showing no significant gender differences in the relationship between the impostor phenomenon and SA. This discrepancy in findings may stem from cultural background differences: in Western individualistic cultures, gender roles and emotional expression are more differentiated, whereas in the collectivist culture of China, the pressure of social evaluation exerts a universal impact on both genders, potentially masking gender’s moderating effect. These findings highlight the importance of considering cultural context when examining gender difference in the psychological consequences of impostor phenomenon.

## 6 Implication

### 6.1 Theoretical implication

This research highlights the relevance of self-compassion and shame in understanding the association between impostor phenomenon and SA. Although the relationships among these variables have been previously established, this study extends existing understanding by examining how the impostor phenomenon, as a deep psychological structure involving self-cognition and self-evaluation, relates to self-compassion, shame, and SA among college students, thereby establishing a systematic theoretical link. The construction of this model provides an integrated theoretical perspective for understanding how individuals’ cognitive beliefs manifest in behavioral patterns and psychological states within social contexts through internal emotional processes. Within the framework of CAPS, this model expands the understanding of the correlational structure of SA from a singular focus on self-evaluation or emotional reaction to an integrated perspective that considers how both sets of factors co-occur with SA. The findings advance understanding underlying the development of SA among college students. They also identify clear potential targets for intervention and highlight the need for a systematic approach that concurrently addresses both cognitive restructuring and emotional regulation in such interventions.

### 6.2 Practical implication

The study puts forward some suggestions for future mental health education and psychological resilience cultivation among college students. Based on the research findings, subsequent preventive and developmental intervention measures should focus on constructing a multi-level, systematic positive psychological adaptation system for college students, thereby fostering intrinsic growth capabilities.

As a college student, an individual should first enhance psychological awareness of the impostor phenomenon. Given the roles of self-compassion and shame proneness, and their association, individual coping efforts should prioritize the cultivation of self-compassion as a foundational step. When experiencing SA or self-doubt triggered by impostor phenomenon, one can consciously initiate self-compassion training methods, such as mindfulness meditation or self-acceptance journaling, to gradually alleviate and reduce the shame and psychological burden associated with it. Building on this, individuals may attempt to consciously and progressively engage in activities within low-pressure, supportive social settings and practice moderate self-disclosure among trusted peers or groups. Through positive feedback and acceptance experiences gained in reality, internal fears can be gradually dissolved, thereby reducing the tendency for social avoidance driven by fear of evaluation.

From a teacher’s perspective, efforts should be made to guide students in recognizing and scientifically understanding the impostor phenomenon. This can be achieved through classroom discussions, case-based teaching, mental health workshops, and other formats to help students recognize the commonality of this psychological experience during academic development and growth stages. In teaching practices, teachers should consciously incorporate brief self-compassion exercises, such as five-minute mindful breathing or reflective writing, to help students cultivate self-compassion. In classroom interactions and feedback, teachers can de-emphasize overly competitive atmospheres, highlighting growth in the learning process rather than mere outcome comparisons, to alleviate students’ feelings of shame. Additionally, teachers should remain attentive to students’ psychological states, particularly those exhibiting excessive caution, avoidance of self-expression, or extreme anxiety about evaluation, encouraging them to gradually participate in social activities while providing non-judgmental support.

At the institutional level, the association revealed by this study suggests the value of establishing a hierarchical support system, which should simultaneously focus on protective factors and vulnerability factors. Universities can integrate knowledge about the impostor phenomenon and coping strategies into mandatory mental health education curricula and disseminate related information through workshops, themed lectures, and other formats. The potential value of self-compassion exercises in alleviating feelings of shame and SA should be highlighted. For students who already exhibit signs of shame-driven social avoidance, counseling centers could offer workshops or group sessions based on the principles of Compassion-Focused Therapy, an approach specifically designed to address shame proneness. Simultaneously, academic evaluation systems could be adjusted to de-emphasize single competitive indicators and incorporate process-oriented, growth-based assessment dimensions, thereby institutionally alleviating students’ pressure to prove their competence. Furthermore, universities should strengthen relevant teacher training and support the implementation of peer support programs, encouraging students to engage in self-disclosure and experience-sharing within safe and accepting group environments.

### 6.3 Limitations and future directions

This study provides a preliminary exploration of the internal connection between impostor phenomenon and SA, offering significant insights into the association between psychological experiences and social adaptation challenges in college students. While acknowledging the contributions of this research, it is essential to objectively examine its limitations. Firstly, the cross-sectional nature of the research design remains a key limitation. Although hierarchical regression analysis tests the theoretical relationship between variables, it neither permits causal inferences nor establishes temporal precedence, as the cross-sectional design precludes empirical verification of causal sequences or reciprocal relationships, despite certain directional assumptions implied in the hypothesized model. To further clarify the trajectories of mutual influence among variables, future research should adopt longitudinal designs with multi-wave data collection to test the directions of effects. Secondly, the issue of omitted variable bias was not addressed in this study. Potentially relevant variables such as depression, neuroticism, and trait anxiety, which are closely associated with both shame and SA, were not included in the analytical model. Their absence may obscure the unique predictive effects of the impostor phenomenon and should be considered in future research to strengthen causal inference and model completeness. Third, this study focused primarily on cognitive and emotional factors at the individual level and did not systematically examine the moderating effects of external environmental factors, such as family expectations, campus competitive culture, or peer feedback. Future research could integrate macro-environmental contexts with micro-psychological processes to construct more comprehensive, multi-level theoretical models. Finally, the sample of this study consists entirely of Chinese college students, which helps us to gain an in-depth understanding of psychological correlates and patterns within a specific cultural context. However, considering the significant differences between the collectivist orientation of Chinese culture and the individualist orientation of Western cultures, these cultural values may limit the generalizability of the research findings. Future research should recruit samples from diverse cultural backgrounds (including Western countries) to conduct cross-cultural comparisons, in order to examine the boundary conditions and cross-cultural validity of the conclusions drawn from this study.

## 7 Conclusion

This study, situated within the context of psychological adaptation challenges in contemporary higher education, systematically examines the associations among college students’ impostor phenomenon and SA. The findings manifest a significant positive correlation between impostor phenomenon and SA, which remained significant even after accounting for the unique contributions of self-compassion and shame. Gender significantly moderates the associations between impostor phenomenon and both self-compassion and SA. These findings shed light on the complex psychological interrelations connecting the impostor phenomenon and SA in college students. These results offer the systematic theoretical foundation and feasible practical pathways for forestalling and intervening in SA within this population. Notably, while the cross-sectional design employed in this study reveals correlational patterns among variables, it limits causal inferences regarding the direction of effects. Future research could utilize time series data or controlled intervention experiments targeting key variables to further examine the causal pathways and dynamic influence mechanisms among these factors.

## Data Availability

The datasets presented in this study can be found in online repositories. The names of the repository/repositories and accession number(s) can be found in the article/supplementary material.
